# Rates of Mutations and Transcript Errors in the Foodborne Pathogen *Salmonella enterica* subsp. *enterica*

**DOI:** 10.1093/molbev/msac081

**Published:** 2022-04-21

**Authors:** Jiao Pan, Weiyi Li, Jiahao Ni, Kun Wu, Iain Konigsberg, Caitlyn E. Rivera, Clayton Tincher, Colin Gregory, Xia Zhou, Thomas G. Doak, Heewook Lee, Yan Wang, Xiang Gao, Michael Lynch, Hongan Long

**Affiliations:** 1 Institute of Evolution and Marine Biodiversity, KLMME, Ocean University of China, 5 Yushan Road, Qingdao, Shandong Province 266003, China; 2 Laboratory for Marine Biology and Biotechnology, Qingdao Pilot National Laboratory for Marine Science and Technology, Qingdao 266237, China; 3 Department of Biology, Indiana University, Bloomington, IN 47405, USA; 4 Division of Biomedical Informatics & Personalized Medicine, Department of Medicine, University of Colorado, Aurora, CO 80045, USA; 5 National Center for Genome Analysis Support, Indiana University, Bloomington, IN 47405, USA; 6 School of Computing and Augmented Intelligence, Arizona State University, Tempe, AZ 85281, USA; 7 State Key Laboratory of Microbial Technology, Microbial Technology Institute, School of Life Science, Shandong University, No. 72 Binhai Road, Qingdao, Shandong Province 266237, China; 8 Biodesign Center for Mechanisms of Evolution, Arizona State University, Tempe, AZ 85281, USA

**Keywords:** *Salmonella*, spontaneous mutation, bacteria, genome evolution, transcript error

## Abstract

Because errors at the DNA level power pathogen evolution, a systematic understanding of the rate and molecular spectra of mutations could guide the avoidance and treatment of infectious diseases. We thus accumulated tens of thousands of spontaneous mutations in 768 repeatedly bottlenecked lineages of 18 strains from various geographical sites, temporal spread, and genetic backgrounds. Entailing over ∼1.36 million generations, the resultant data yield an average mutation rate of ∼0.0005 per genome per generation, with a significant within-species variation. This is one of the lowest bacterial mutation rates reported, giving direct support for a high genome stability in this pathogen resulting from high DNA-mismatch-repair efficiency and replication-machinery fidelity. Pathogenicity genes do not exhibit an accelerated mutation rate, and thus, elevated mutation rates may not be the major determinant for the diversification of toxin and secretion systems. Intriguingly, a low error rate at the transcript level is not observed, suggesting distinct fidelity of the replication and transcription machinery. This study urges more attention on the most basic evolutionary processes of even the best-known human pathogens and deepens the understanding of their genome evolution.

## Introduction

*Salmonella enterica* subsp. *enterica* (subsequently referred to as *S. enterica*) is one of the major bacterial food-poisoning pathogens, causing frequent gastroenteritis outbreaks and tens of thousands of deaths per year, and is closely monitored by disease control and prevention agencies of most developed countries. As a model microorganism, this species is widely used in various biological studies and for developing numerous bio-techniques, such as gene editing, Ames mutagenicity testing, phenotypic, and molecular detection, etc. Genomic and evolutionary research has also provided deep insights into the virulence, host adaptation, and drug resistance of *S. enterica* (Parkhill et al. 2001; Roumagnac et al. 2006; Holt et al. 2008; Langridge et al. 2015).

One recent pan-genome study using ancient and modern *S. enterica* DNA sequences showed that only a few genomic changes occurred in a lineage over about 3000 years, implying an unusually low evolutionary rate compared with other bacteria (Zhou et al. 2018). It has also long been felt that *S. enterica* is less prone to mutations than *Escherichia coli* when treated with UV or other DNA-damaging agents, perhaps because of the higher replication fidelity of DNA polymerase V during the SOS response (Koch et al. 1996; Kokubo et al. 2005). Also differing from many other bacteria, *S. enterica* is thought to have a low level or no recombination, especially within non-Typhimurium lineages (Didelot et al. 2011). Nonetheless, one pioneering mutation-accumulation study on the model strain—*S.* Typhimurium LT2, reported a spontaneous mutation rate of ∼0.003 per genome per generation (9), a quite high mutation rate among DNA-repair-functional bacteria studied with deep whole-genome sequencing (table 1, blue). However, because only one wild-type mutation-accumulation line with 4.9× depth of sequencing coverage was evaluated in this study, when most advanced bioinformatic tools had not been developed, the results were thus affected by false positives as pre-warned by the authors (Lind and Andersson 2008); a more thorough evaluation of the rate and molecular spectrum of mutation is needed to systematically evaluate the genome stability of *S. enterica*. Because within-species mutation rate/spectrum variation is not uncommon (Ness et al. 2015; Long, Behringer et al. 2016), such a study ought to involve multiple strains of different origins. A variance of *de novo* mutations could help to understand mutation-rate determinants, how mutations contribute to genome architecture evolution between closely related lineages, address the evolutionary potential of populations, and so on (Francioli et al. 2015; Ness et al. 2015; Lynch et al. 2016; López-Cortegano et al. 2021). Despite this, genome-wide studies on *de novo* mutation-rate variance within-species, especially for unicellular organisms including *S. enterica*, have been extremely rare and possibly barriered by the tremendous amount of resources and time required for the experiments and analyses (Ness et al. 2015; Wu et al. 2021). Notably, the evolutionary mechanisms of pathogenicity genes are still unclear, and whether such a variation that is associated with unusual mutational features is also unknown (Galán 2009; Galán et al. 2014; Journet et al. 2016; Gao et al. 2017).

**Table 1.**
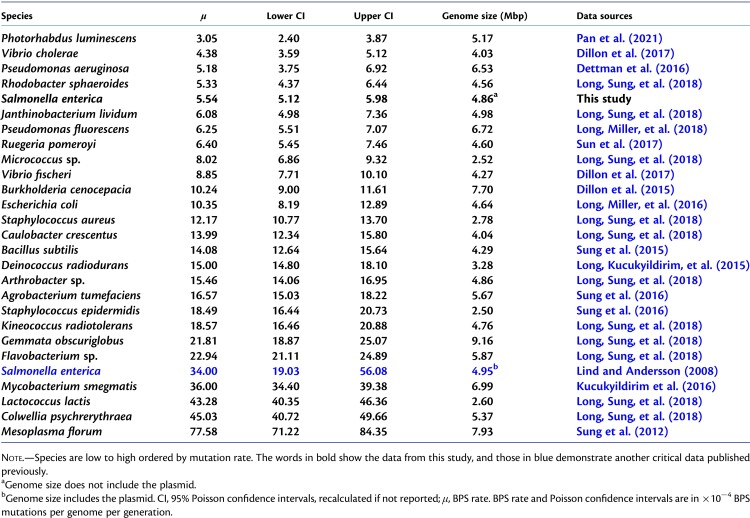
The Base-Pair Substitution (BPS) Mutation Rates of Bacteria Estimated with MA-WGS.

Thus, to unbiasedly evaluate the spontaneous mutations of this important pathogen, we performed large-scale mutation-accumulation experiments combined with deep whole-genome sequencing (MA-WGS) on 18 *S*. *enterica* strains of eight serovars and from world-wide collection sites, including three DNA mismatch repair (MMR) knockout strains (table 2, supplementary dataset S1: table S1, Supplementary Material online). For each strain, a large number of parallel MA lines were initiated from a single ancestral cell and cultured on rich and nonstressing media. Each replicate subline was then repeatedly bottlenecked by single-individual transfers for thousands of generations, providing a setting in which genetic drift dominates selection and ample material for obtaining refined resolution of the mutational landscape (Bateman 1959; Mukai and Yamazaki 1964; Lynch et al. 2008; Halligan and Keightley 2009). Because spontaneous mutations are unbiasedly fixed in each MA line, the intrinsic fidelity of replication machinery and efficiency of the DNA mismatch repair system are revealed.

**Table 2.** General Information of *S. enterica* in This Study.

Serovar	Strain	Catalog No.	*n*	cov	Sites	*G*	BPS	*m*	ts/tv	Indels	Errors	Ne
Agona	BAA-707(T)	BAA-707	43	113	4.38	2034	54	1.66	1.57/8.46	9(7,2)	700(3)	14
Bareilly	9115(H)	ATCC9115	45	83	4.46	2037	50	2.90	0.79/5.74	11(8,3)	1470(3)	14
Dublin	2469(Q)	SGSC2469	42	88	4.45	2040	47	4.14	1.61/7.09	6(4,2)	977(3)	14
Enteritidis	LJH608(I)	BAA-1045	43	76	4.40	1960	47	2.56	2.36/7.92	2(2,0)	784(3)	13.5
Newport	C487-69(P)	ATCC27869	40	96	4.49	2118	50	4.20	1.17/6.49	4(3,1)	2037(3)	14.5
Paratyphi A	9150(J)	ATCC9150	43	106	4.22	2031	45	2.21	1.25/5.18	3(3,0)	1402(3)	13.5
Typhi	ST1(D)	SGSC2728	44	125	4.24	1988	36	1.53	1.12/6.30	3(1,2)	1686(3)	13.5
Typhi	CT18(E)	SGSC4072	41	98	4.24	1984	49	1.57	1.58/5.80	5(2,3)	1924(4)	13.5
Typhi	Ty2(F)	SGSC2666	44	116	4.24	1946	35	3.10	1.06/6.39	6(2,4)	1405(3)	13
Typhi	PNG31(G)	SGSC3194	44	121	3.52	1984	23	1.20	0.92/6.11	8(5,3)	1543(3)	13.5
Typhi	CDC3137-73(L)	SGSC2660	44	123	4.25	1970	39	1.29	1.05/6.39	3(3,0)	1802(3)	13.5
Typhi	CDC1707-81(M)	SGSC2661	45	116	4.25	1954	42	1.74	1.33/5.58	6(4,2)	856(3)	13.5
Typhi	CDC382-82(N)	SGSC2664	42	131	4.20	1994	28	1.63	6.00/5.74	8(6,2)	1422(3)	14
Typhi	CDC9228-77(R)	SGSC2657	42	127	4.13	1923	20	2.70	5.67/5.51	4(1,3)	1420(4)	13
Typhimurium	LT2(K)	ATCC19585	47	139	4.81	2000	80	2.34	1.22/5.59	6(4,2)	712(2)	13.5
Typhimurium	LT2 Δ*mutH*(C)	SGSC1350	12	99	4.81	2079	4287	0.56	70.45/–	220(54,166)	–	14
Typhimurium	LT2 Δ*mutL*(A)	SGSC1348	10	94	4.80	2002	2817	0.81	60.24/–	186(65,121)	–	13.5
Typhimurium	LT2 Δ*mutS*(B)	SGSC1349	9	254	4.81	2002	2349	0.67	64.25/6.65	183(59,124)	130(2)	13.5

note.—BPSs, total number of base-substitution mutations in all MA lines; Catalog No., catalog numbers from American Type Culture Collection (strain name starting with ATCC or BAA) or *Salmonella* Genetic Stock Center (starting with SGSC); cov, mean depth of sequencing coverage; *G*, mean number of generations of MA lines; Errors, total number of transcript errors, number of replicates is in parentheses; Indels, total number of indel mutations in all MA lines; *m*, mutation bias (*μ*_G/C→A/T_/*μ*_A/T→G/C_) in the A/T direction; *n*, number of MA lines; Sites, mean number of sites with reads covered, in Mbp; Strain, strain names with MA group letter in parentheses; ts/tv, the ratios of transition to transversion mutations/transcript errors; *N_e_*, the effective population size of the MA lines, estimated by the harmonic mean method.

Compared with extensive studies on DNA errors, that is, genomic mutations, very few studies have focused on RNA errors at the level of transcripts because of technical hurdles to accurately detect *in vivo* transcript errors. With the recent development of the rolling-circle amplification-based sequencing (CirSeq) method (Lou et al. 2013; Traverse and Ochman 2016; Gout et al. 2017; Fritsch et al. 2021), cross-species comparisons of transcript errors became available. However, a variance of the transcript-error rate and molecular spectrum within one species remains unexplored, which requires many strains each with multiple replicates for highly accurate estimates. Therefore, in parallel with the MA-WGS approach to investigate spontaneous mutations, CirSeq libraries were constructed for the ancestral strains to accurately identify transcript errors and evaluate whether there is a within-species variation in the rate and molecular spectrum of transcript errors. Such explorations could also help to develop the biophysical models for cellular stability and test transcription-associated hypotheses.

## Results

To quantitatively evaluate the Genomic Mutation rate and reveal within-species mutation-rate variation, we ran MA experiments on 18 *S. enterica* strains, with a total of 768 MA lines (48 MA lines for each of the 15 MMR-functional strains, performed in two batches; 16 for each of the three MMR-dysfunctional strains). Summing over all sublines, a total of 1,359,047 generations was involved (table 2, supplementary dataset S1: table S1, Supplementary Material online). All final MA lines and the corresponding ancestral lines of each strain were subjected to deep Illumina PE150 genome sequencing (supplementary dataset S1: tables S2–S19, Supplementary Material online), leading to a 116.87× (SE: 9.06) mean depth of sequencing coverage, and an average 90.02% (SE: 1.53%) of the genome covered with high-quality reads, using the chromosome sequence of the type and model strain *S.* Typhimurium LT2 as the reference. Experimental details are listed in table 2, Materials and Methods, and supplementary dataset S1: tables S2–S19, Supplementary Material online. Since lethal or heavily deleterious mutations could be lost even under the extreme bottleneck effect of MA transfers and if mean fitness costs of mutations are high, mutation accumulation could be biased by purifying selection. To test this, for the MA dataset of each strain, we compared the nonsynonymous:synonymous mutations ratio with the random expectation—nonsynonymous:synonymous mutation sites ratio of the genome, and did not detect any sign of mutations being biased by purifying selection in any strain (supplementary dataset S1: table S20, Supplementary Material online); the mean effective population size (*N_e_*) was ∼14, confirming again that genetic drift dominated selection during the experiments (table 2).

**Fig. 1.**
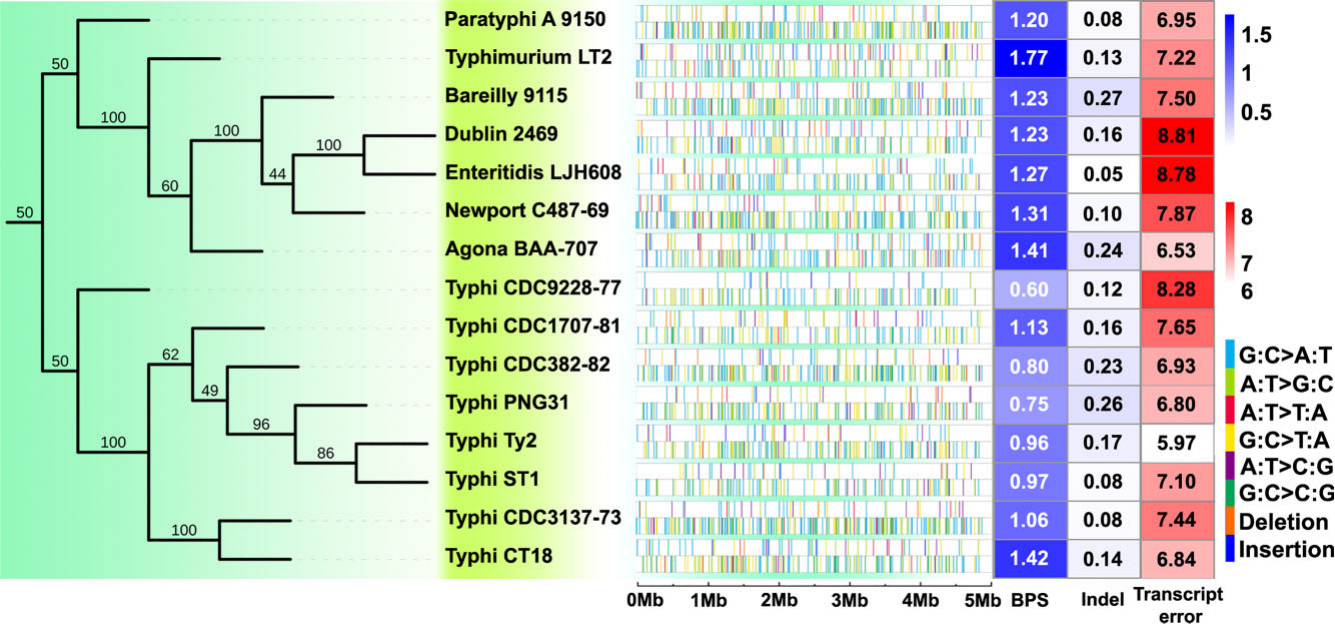
Rates and distribution of mutations and transcript errors of the 15 MMR-functional *Salmonella enterica* strains. (Left) The phylogenetic relationship of the strains based on whole-genome SNPs. (Center) The genome-wide distribution of mutations for each strain (the upper row with sparse colored bars) and transcript errors (the lower row with dense colored bars). (Right) Heat map showing for each strain BPS mutation rates × 10^−10^ per site per generation; indel mutation rates × 10^−10^ per site per generation; and transcript-error rates × 10^−6^ per site per transcription.

**Fig. 2.**
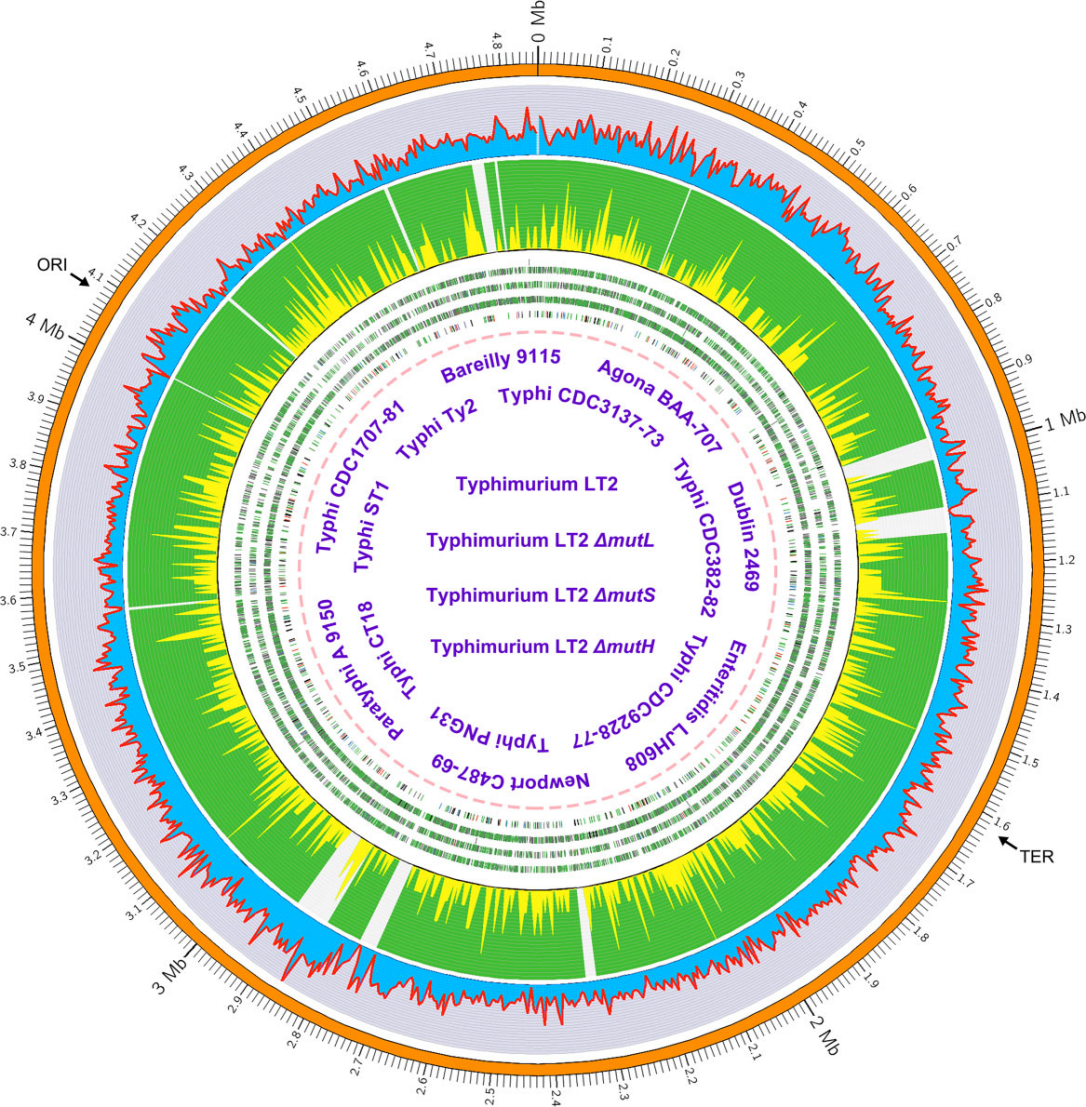
Genome-wide distribution of mutations. Circles from outside to inside (different colors of lines and backgrounds are only for contrast purposes): orange, genomic coordinates in Mbp; blue with red outlines, the number of mutations per 4.86-kbp bin (the whole genome contains 1,000 bins), with mutations pooled from MA lines of all three MMR-deficient *S.* Typhimurium LT2 strains (Δ*mutS*, Δ*mutL*, Δ*mutH*); green with yellow bars, the number of mutations per 4.86-kbp bin, mutations are pooled from MA lines of all 15 MMR-functional strains (table 2), the gray blocks in the green circle represent regions without reads covered in most non-LT2 wild-type strains; the genomic distribution of mutations in *S.* Typhimurium LT2 Δ*mutL*, Δ*mutS*, Δ*mutH*, all the MMR-functional strains (the four groups of colored tiles from outside to inside)—red, gray, blue, green, purple, and black tiles mark different types of base-substitution mutations—A:T → C:G, A:T → G:C, A:T → T:A, G:C → A:T, G:C → C:G and G:C → T:A, respectively, note that five single tiles were jittered from the tiles below due to overlapping or being too close with other mutations. ORI, origin of replication; TER, replication terminus.

To evaluate transcript errors, we applied a modified CirSeq library construction protocol and Illumina SE300 sequencing on 72 RNA samples (four replicates for each of the 18 strains), generating an average of 5.69 × 10^7^ (SE: 0.58 × 10^7^) bases per sample. After applying quality-control filters and preliminary analyses, 680 total MA lines and 48 CirSeq samples for transcript-error analyses were used for final analyses (table 2, supplementary dataset S1: tables S2–S19, S21, Supplementary Material online), with library-failed and/or low depth-of-coverage lines excluded (supplementary dataset S1: table S22, Supplementary Material online). The details of all base-pair substitutions (BPS), small indels, and transcript errors are shown in figure 1 and supplementary dataset S1: tables S23–S25, Supplementary Material online, and their genome-wide distribution is outlined in figure 2.

### Low Genomic Mutation Rate

**Fig. 3.**
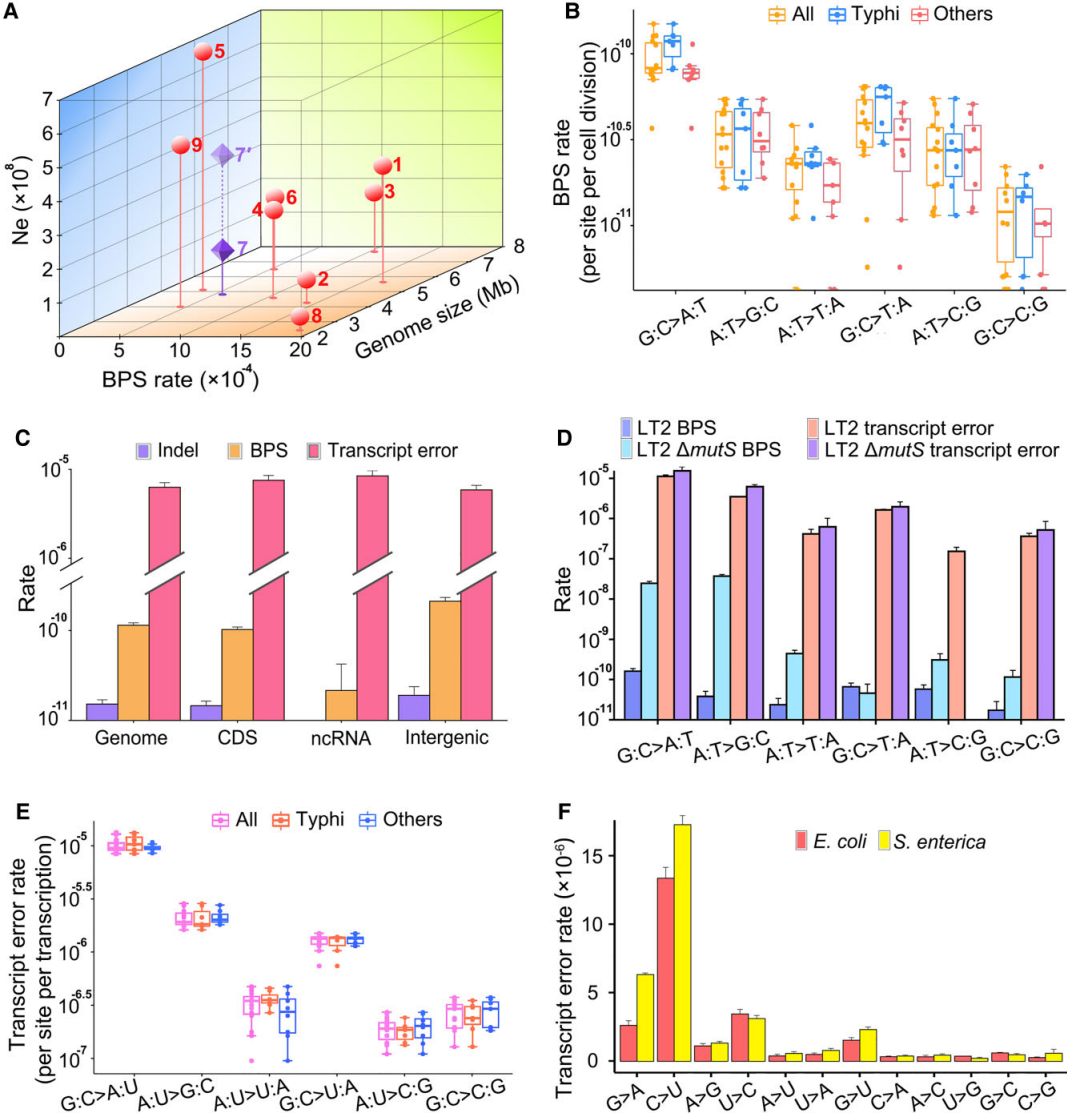
Mutation and transcript-error comparison between different genomic regions, strains, or species. (*A*) The genome size, base-substitution rate (per genome per generation) and effective population size (*N_e_*) of bacteria studied with MA-WGS (Dettman et al. 2016; Lynch et al. 2016; Long, Sung, et al. 2018; Pan et al. 2021). 1—*Agrobacterium tumefaciens* C58; 2—*Bacillus subtilis* NCIB 3610; 3—*Burkholderia cenocepacia* HI2424; 4—*Escherichia coli* K-12 MG1655; 5—*Photorhabdus luminescens* ATCC29999; 6—*Pseudomonas aeruginosa* PA14; 7—*Salmonella enterica* (dark symbol uses *N_e_* from this study; 7′ light symbol uses *N_e_* from Bobay and Ochman, 2018); 8—*Staphylococcus epidermidis* ATCC 12228; 9—*Vibrio cholerae* 2740-80. (*B* and *E*) Mutation and transcript-error spectra of the MMR-functional strains; all—refers to all the strains; Typhi—eight strains of the Typhi serovar; others—represents seven non-Typhi strains. (*C*) The comparison of BPS mutation rates, indel mutation rates, and transcript-error rates of all MMR-functional strains; CDS, coding sequences; ncRNA, noncoding RNA; Intergenic, genomic regions with coding sequences and noncoding RNA excluded. (*D*) The spectra of mutations and transcript errors in MMR-functional versus Δ*mutS* Typhimurium LT2. (*F*) The molecular spectra (per site per transcription) of transcript errors in the coding sequences of *E. coli* K-12 MG1655 versus *S. enterica—*based on transcript errors pooled from the 15 MMR-functional strains. All error bars are standard errors of the mean.

Genomic mutation rates of the 15 world-wide MMR-functional strains are shown in figure 1 and supplementary dataset S1: tables S2–S16, Supplementary Material online. Analysis of variance (ANOVA) of the among-line variance of the genomic mutation rate reveals significant differences (ANOVA: *F* = 2.82, *P* = 0.0004). A further Tukey test shows that the mutation rate of Typhimurium LT2 is significantly higher than those of another three Typhi strains (Typhi CDC9228-77: *P* = 0.0003; Typhi PNG31: *P* = 0.0034; Typhi CDC382-82: *P* = 0.0087; fig. 1). Thus, the mutation rate varies within *S. enterica*. The accumulated mutations across all 15 strains yield a mean BPS mutation rate of 1.14 × 10^−10^ per nucleotide site per generation (SE: 0.08 × 10^−10^), or 5.54 × 10^−4^ (SE: 0.40 × 10^−4^) BPS mutations per genome per generation, one of the lowest mutation rates among studied bacteria (table 1 and fig. 3*A*). Because *S.* Typhi specifically infects human hosts, causing typhi fever, we wondered whether Typhi strains show differences in mutational features relative to those in other serovars. Eight of the 15 MMR-functional strains that we studied are of the Typhi serovar, either model or natural ones (table 2 and fig. 3*B*, supplementary dataset S1: tables S8–S15, Supplementary Material online). There is no significant difference in the overall mutation rates of Typhi strains (ANOVA, *F* = 2.07, *P* = 0.05; fig. 1).

Because the Type III secretion system of *S. enterica* plays a major role in virulence, enabling the injection of effector proteins into the cytoplasm of host cells, we specifically queried whether the genes of the *Salmonella* pathogenicity islands (SPI—genomic “islands” containing the virulence genes) (Blanc-Potard et al. 1999; Hensel 2000; Lostroh and Lee 2001) spontaneously mutate at different rates from other genomic regions. To test this, genes hit with BPSs in MMR-dysfunctional *S.* Typhimurium LT2 strains (Δ*mutS*, Δ*mutL*, Δ*mutH*) were pooled to increase statistical power (supplementary dataset S1: table S26, Supplementary Material online). We found no difference in the incidence of mutations in SPI versus non-SPI genes, 3.82 × 10^−8^ (CI: 3.15 × 10^−8^–4.58 × 10^−8^) versus 3.78 × 10^−8^ (3.70 × 10^−8^–3.86 × 10^−8^) per site per generation, respectively (Mann–Whitney *U* test, *P* = 0.26). Thus, SPI evolution is not accelerated by mutation-rate inflation. To find out other evolutionary forces shaping the SPI evolution of natural strains, we also retrieved pathogenicity genes of 265 *S. enterica* natural strains with complete genomes from the NCBI Genome database (supplementary dataset S1: table S27, Supplementary Material online) and calculated dN/dS (the ratio of nonsynonymous to synonymous SNP rates) of each gene. The mean dN/dS is 0.38 (SD: 0.18; excluding the outlier gene SseD-Secretion system effector D, with dN/dS ∼ 16; supplementary dataset S1: table S28, Supplementary Material online), consistent with previous estimates on all genes in the genome (0.66 ± 0.05) (Holt et al. 2008), supporting purifying selection. All the above demonstrates that pathogenicity genes generally do not exhibit elevated mutation rates and are under purifying selection.

Among the mutation spectra of the 15 MMR-functional strains, we find significant differences in G:C → A:T transition rates between Typhimurium LT2 and Typhi PNG31 (ANOVA, *F* = 1.80, *P* = 0.04; Tukey test, *P* = 0.02) (fig. 3*B*), indicating that there is a within-species variation in mutation spectrum. The mutation spectrum based on the pooled BPS mutations of these strains, shown in figure 3*B*, has features similar to those documented for many other bacteria, including: (1) transitions dominating transversions (transition to transversion ratio: 1.91, SE: 0.42); (2) mutations being biased in the A/T direction (mutation bias-μ_G/C→A/T_/μ_A/T→G/C_: 2.32, SE: 0.41), consistent with the A/T mutation bias found in most other bacteria; and (3) methylated sites do have elevated mutation rate (supplementary dataset S1: table S29, Supplementary Material online) (Lynch and Walsh 2007; Hershberg and Petrov 2010; Lee et al. 2012). The BPS mutation rate of 1.02 × 10^−10^ (SE: 0.07 × 10^−10^) in coding regions is significantly lower than the 2.11 × 10^−10^ (SE: 0.22 × 10^−10^) observed in intergenic regions (fig. 3*C*), which indicates that coding regions are less susceptible to mutations than intergenic regions, consistent with the observation in *Escherichia coli* (Lee et al. 2012) (supplementary dataset S1: table S30, Supplementary Material online). The nonsynonymous/synonymous mutation ratios of most strains are not significantly different from the random expectations (supplementary dataset S1: table S20, Supplementary Material online), and thus, there is no sign of purifying selection biasing the coding-region mutation rate. To exclude the possibility that this is caused by differences in nucleotide composition, we normalize the mutation rates by taking the GC contents of different regions into consideration (Long, Sung, et al. 2015); the BPS mutation rates are then 9.81 × 10^−11^ (CI: 8.96 × 10^−11^–1.07 × 10^−10^) and 2.25 × 10^−10^ (CI: 1.90 × 10^−10^–2.65 × 10^−10^) in coding and intergenic regions, respectively. Intergenic regions are indeed more mutable than coding regions. This might be associated with the possible transcription-associated DNA repair systems and needs further exploration.

The overall mutation rate for small indels is estimated to be 0.15 × 10^−10^ (SE: 0.02 × 10^−10^), about 13% of the BPS rate, with a bias to deletions (insertion bias *μ*_insertion rate_/*μ*_deletion rate_: 0.80) (table 1, supplementary dataset S1: table S31, Supplementary Material online). Knocking out the MMR decreases the indel-rate/BPS-rate ratio to 6.38%, inferring that the MMR preferentially repairs BPS than the indels. There is no significant indel-rate difference among strains (ANOVA, *F* = 1.24, *P* = 0.24); 36.90% of indels occur in simple sequence repeats (SSR) regions for all the MMR-functional strains, whereas 95.25% for MMR-deficient strains do so, that is, the indels are prone to be repaired in repeat regions (supplementary dataset S1: table S24, Supplementary Material online). In contrast to the BPS mutations, the indels do not show locational bias with respect to coding versus intergenic regions (fig. 3*C*, supplementary dataset S1: table S30, Supplementary Material online).

### Influence of DNA Mismatch Repair on Mutations

Comparing the mutational features of MMR^+^ and MMR^−^ strains helps to reveal the specificity of DNA repair and mutagenesis bias of premutations (mutations prior to the DNA repair) (Long, Miller, et al. 2018). To study this, MA experiments were performed with the MMR^+^*S.* Typhimurium LT2 strain and another three MMR-dysfunctional strains (Δ*mutS*, Δ*mutL*, Δ*mutH*) derived from LT2, where 80, 2349, 2817, 4287 BPSs were detected, respectively (table 2). Similar to all studied bacteria, the mutation rates of MMR-deficient strains follow a wave-like distribution, with the highest mutation rates around 3 and 9 o’clock of the genome and lowest at the origin of replication, nucleotides with flanking G or C contexts do have elevated mutation rate than those without, for example, the A flanked with G and C could be ∼47× higher in mutation rate than the G flanked by A and T (fig. 2, supplementary dataset S1: table S32, and figs. S1, S2, Supplementary Material online) (Lee et al. 2012; Foster et al. 2013; Long, Sung, et al. 2015; Sung et al. 2015).

As in most other studied bacteria, the MMR of *S*. Typhimurium LT2 preferentially repairs transitions, especially A:T → G:C (fig. 3*D*): MMR repair-efficiencies for transitions and transversions (calculated by (Δ*mutS* mutation rate − wild-type mutation rate)/Δ*mutS* mutation rate) are 99.65% and 81.18%, respectively, and the repair efficiency for the A:T → G:C transitions is the highest—99.88% (supplementary dataset S1: table S33, Supplementary Material online). As a consequence, knocking out the MMR elevates the transition to transversion ratio by ∼34× (fig. 3*D*, supplementary dataset S1: table S33, Supplementary Material online). The overall BPS mutation rate under the MMR deficiency is about 174× higher than under the MMR functionality, that is, 0.58% (SE: 0.06%) of the premutations escape the MMR. This is a relatively high MMR repair efficiency among bacteria, for example, 0.94% (SE: 0.11%) in *E. coli*, and knocking out the MMR elevates its mutation rate by 104× (Long, Miller, et al. 2018). 0.52% (SE: 0.06%) BPS escaping the MMR in coding regions is slightly lower than 1.08% (SE: 0.28%) in intergenic regions, demonstrating again the low susceptibility of coding regions to base-substitution mutations at the DNA repair level.

For indels, the MMR deficiency elevates the mutation rate by a factor of 148×, implying the repair of ∼99.32% (SE: 0.28%) of all preindel mutations by the MMR. Similar to the findings in most other studied bacteria, the MMR also repairs insertions at slightly higher efficiency than deletions (0.33% escape MMR, SE: 0.23% vs. 1.47%, SE: 0.74%) (Long, Miller, et al. 2018). The indels falling in SSRs are repaired at an efficiency of 99.76% (SE: 0.17%), higher than those in the non-SSR regions, 90.43% (SE: 5.11%). The indels falling in coding regions are repaired at similar efficiency to those in the intergenic regions (98.33%, SE: 0.84% vs. 98.95%, SE: 0.75%), a pattern different from that of the BPSs.

### A Low Error Rate at the Transcript Level is Not Observed

From the successfully sequenced 48 CirSeq samples of 16 strains, we detected a total of 20,270 transcript errors (table 2, supplementary dataset S1: table S25, Supplementary Material online). Transcript errors of the 15 MMR-functional strains yield a mean transcript-error rate estimate of 7.38 × 10^−6^ per site per transcription, without a significant within-species variation (SE: 0.21 × 10^−6^; ANOVA: *F* = 0.93, *P* = 0.54; fig. 3*E*). The gene-specific transcript-error rates do not correlate with the expression level, consistent with the mutation rate versus the expression level (supplementary fig. S3, Supplementary Material online). We do not detect any correlation between mutations and transcript-error rates across all surveyed loci (*r* = −0.01, *P* = 0.68), nor any sites as hotspots for both mutations and transcription errors (supplementary dataset S1: table S34, Supplementary Material online). Contrary to its low genomic mutation rate (fig. 3*A*), the transcript-error rates are not among the lowest compared with those from previously studied bacteria (Li and Lynch 2020), with G → A and C → U transitions being the main contributors (fig. 3*F*).

Transcript errors of the 15 MMR-functional strains show a mean A/T bias of 5.18 (SE: 0.34), much higher than that of mutations ∼2.32, and mainly resulting from the extremely abundant G → A and C → U transcript errors (fig. 3*B* and *E*). Except for G/C → A/T(U) transitions being the most dominant mutation/transcript-error types, the transcript-error spectrum is highly different from that of mutations (fig. 3*B*, *E*, and *F*), clearly confirmed by the contrasting transition/transversion ratios: 6.29 (SE: 0.24) versus 1.91 (SE: 0.42) for the transcript errors and mutations, respectively. In terms of functional context, the coding regions and structural RNAs do not show any significant difference in the transcript-error rates, whereas that of other transcribed regions such as UTRs is slightly lower (supplementary dataset S1: table S30, Supplementary Material online).

## Discussion

This massive study directly reveals the low mutation rate of the important foodborne pathogen *S*. *enterica* and sheds light on its long-term evolution; however, the revealed error rate at the transcript level is not accordingly low. Based on the genomic mutation patterns of 15 MMR-functional strains of diverse serovars, our rate estimate, which is only ∼1/6 of that previously reported for a single *S*. Typhimurium LT2 strain in a much smaller study (table 1) (Lind and Andersson 2008), provides direct evidence for the relatively high genome stability of *S. enterica* among bacteria (fig. 3*A* and table 1). By using the false-positive rate ∼53% reported by Lind and Andersson (2008), we calibrated the LT2 mutation rate to be 3.71 × 10^−10^ per site per cell division or 0.0016 per genome per cell division, about twice higher than the one reported in this study: 1.77 × 10^−10^ and 0.00086. Based on the calibrated mutation rate, we further calculated the Poisson confidence intervals—(1.48 × 10^−10^, 7.59 × 10^−10^), which do overlap with the ones reported in this study—(1.40 × 10^−10^, 2.20 × 10^−10^).

A comparison of mutation patterns between MMR-functional and -dysfunctional *S.* Typhimurium LT2 strains also shows that the high MMR-efficiency is one major cause of the low mutation rate. Our results are also consistent with previous indirect studies reporting very few pan-genome changes between ancient and modern *S. enterica* lineages in human teeth, high-fidelity DNA polymerases upon stress response, etc. (Koch et al. 1996; Zhou et al. 2018). We note that as in all MA studies using short-read sequencing, reliable structural variants were still not resolved in this study, and future trials using long-read sequencing are required to provide more insight into this issue.

To understand why *S. enterica* has a low spontaneous mutation rate, we first confirmed that the results are not technical artifacts caused by false negatives (see Materials and Methods, testing for false negative mutations), and then, evaluated the effective population size (*N_e_*) of this species, as a high *N_e_* can be conducive to the evolution of low error rates according to the drift-barrier hypothesis (fig. 3*A*) (Dettman et al. 2016; Lynch et al. 2016; Long, Sung, et al. 2018; Pan et al. 2021). For haploid organisms, in mutation-drift equilibrium at neutrally evolving sites, *N_e_* = *π_s_*/2*μ*, where *π_s_* is the pair-wise genetic distance at 4-fold degenerate (silent) sites, and *μ* is the BPS mutation rate per site per generation. Since 4-fold degenerate sites are actually not absolutely neutral, which is indeed the case as shown in previous studies (Shields et al. 1988; Ochman 2003; Chamary et al. 2006; Long, Sung, et al. 2018), the *π_s_* would then be downwardly biased by selection and our *N_e_* calculated here would be a lower limit. Combined with numerous recently sequenced natural strains (e.g., by the US Food and Drug Administration), the refined genomic mutation rate estimated here provides an opportunity to calculate *N_e_* of *S. enterica*. To this end, we retrieved raw reads of 210 natural strains with an intact MMR function, which originated from 46 states of the USA and covered all serovars used in this study, and mapped them to the *S.* Typhimurium LT2 chromosome (supplementary dataset S1: table S35, Supplementary Material online). The all-site (all sites: 4836523; SNPs: 364576) and the silent-site *π_s_* (4-fold degenerate sites: 683211; SNPs: 64658) of *S. enterica* were estimated to be 0.021 and 0.028, with an application of the latter leading to a *N_e_* estimate of 1.24 × 10^8^. One study, using genome assemblies of 400 *S. enterica* strains—different strains from ours—and the mutation rate estimated previously (Lind and Andersson 2008), reported an even higher *N_e_* of 4.03 × 10^8^ (Lind and Andersson 2008; Bobay and Ochman 2018) (fig. 3*A*).

Therefore, the relatively large *N_e_* among bacteria facilitates effective natural selection for antimutators in this species (fig. 3*A*). The targets of such selection might include: the DNA mismatch repair system of *S. enterica*, which is more efficient than those in many other bacteria (Long, Miller, et al. 2018); the DNA polymerases, such as UmuDC, which during stress response in *S. enterica,* has a higher fidelity than those of other bacteria when exposed to UV-induced DNA damage (Koch et al. 1996); other possible unknown antimutator genes, especially in the strains with extremely low mutation rates, such as *S.* Typhi PNG31 and CDC9228-77, in this study (table 2, supplementary dataset S1: tables S11, S15, Supplementary Material online).

*N_e_* of *S. enterica* estimated using the natural strains of multiple serovars in this study—1.24 × 10^8^—is much larger than the *N_e_* for *S.* Typhi: 2.3 × 10^5^ to 1.0 × 10^6^ in (Roumagnac et al. 2006). However, if only using *π_s_* of the 39 natural *S.* Typhi strains and their corresponding mutation rates in our dataset, we calculate *N_e_* to be 4.93 × 10^5^ (*π_s_* = 9.47 × 10^−5^), highly consistent with previous reports for *S.* Typhi, which is known to have diverged from other *Salmonella* lineages quite recently and represents one incipient species (Kidgell et al. 2002; Didelot et al. 2011) (fig. 1, left). However, because of the short divergence time in this recently emergent lineage, these *N_e_* estimates will be downwardly biased, which should reach mutation-drift equilibrium, but have not yet been reached. Thus, the fact that the mutation rate of *S.* Typhi is at the low level found in other *S. enterica* serovars or other facultative pathogens (fig. 3*A*) needs not be inconsistent with the drift-barrier hypothesis, although if true *N_e_* is indeed small, an eventual increase in the mutation rate would be expected. Such possible mutation-rate increase and reduced selection conferred by the small *N_e_* of *S.* Typhi could further elevate the genetic diversity of certain pathogenicity genes, and if so, this might lead to situations in which *S.* Typhi would be more toxic and challenging in future disease treatment.

Not in line with the low mutation rate, a low transcript-error rate of *S. enterica* was not observed compared with previously studied bacteria (Li and Lynch 2020). Given the fairly high error rate at the transcript level, one adaptive hypothesis argues that a high transcript-error rate increases the fitness of an isogenic population facing environmental challenges, such as the antibiotic threat (Meyerovich et al. 2010). However, our transcriptome-wide evaluation of transcript errors reveals that nonsynonymous errors are less frequent than synonymous errors, indicating the nonadaptive nature of transcript errors (*P* = 2.61 × 10^−13^, *χ*^2^ test; supplementary dataset S1: table S36, Supplementary Material online). It remains to be seen whether this fairly high transcript-error rate has been driven by directional evolutionary forces or is simply a result of stochastic drift around the drift barrier. Either way, relatively high transcription-error rates may have relevance to clinical approaches to constraining the success of pathogens, inspiring possible killing strategies by magnifying the transcript-error rate, pushing it over the edge of lethal mutagenesis. To achieve a thorough evaluation of cellular stability at the genome, transcriptome, and proteome levels, future technical advancement in the accurate quantification of translation-error rate is required.

In contrast to the recent advances in the mutational features of nonpathogenic microbes (i.e., biosafety level 1 strains), the studies of human pathogens, especially obligate ones, have barely been explored by mutation-accumulation techniques combining deep whole-genome sequencing. This is mostly because of the technical barriers from limited access to biosafety level 2 or above labs or high costs for numerous lines requiring long-term culturing/genome sequencing. Mutational patterns, particularly the mutation spectra, are known to be influenced by the genome architecture and environmental factors such as pH, antibiotics, etc. (Long, Kucukyildirim, et al. 2015; Long, Miller, et al. 2016; Strauss et al. 2017). Different from opportunistic human pathogens, most obligate ones constantly experience harsh challenges from the immune system, medicines, microbial competitors, etc., and usually have quite streamlined and minimized genomes. Theoretical studies have predicted the elevated mutation rates due to the small *N_e_* (Sung et al. 2012; Lynch et al. 2016), whereas genome-wide mutation spectra are mostly unknown, let alone, how host environments influence their mutagenesis and further affect the pathogenicity evolution. Therefore, systematic evaluation on the genomic mutations of diverse microbial pathogens, each with multiple strains—especially mutation rate in their natural habitats (Zhao et al. 2021)—is urgently needed for testing the generalized mutation-rate evolution hypotheses, understanding their long-term evolution and transmission, as well as guiding infectious-disease treatment and vaccine development at the DNA or RNA levels.

## Materials and Methods

### Strains and MA Experiments

In this study, 18 strains were ordered from the American Type Culture Collection (ATCC, VA, USA) and *Salmonella* Genetic Stock Center (SGSC, University of Calgary, Alberta, Canada); 15 of them were MMR-functional and the remaining three were MMR-knockout (table 2). All the biosafety level 2 procedures were performed under the protocol of 15-038, approved by the Institutional Biosafety Committee, Indiana University at Bloomington. We also sequenced the whole genomes of the ancestral lines of the 18 strains. About 48 and 16 MA lines were initiated for each of the MMR-functional and -dysfunctional strains, respectively; the 48 MA lines for each MMR-functional strain were transferred in two contiguous batches. All the MA lines were cultured on LB agar plates at 37 °C, and repeatedly single-colony transferred, every day for nearly 3 months (table 2). For each strain, we calculated the colony forming units (CFU) using single colonies of five randomly chosen MA lines, every 3 weeks. log_2_(CFU) was then used as the number of cell divisions from a single cell growing to a colony. The grand mean of cell divisions in each colony was calculated for the MA lines of each strain after the MA transfer was finished. The total transfers multiplied by the grand mean were the total number of cell divisions that each MA line passed. After genome sequencing and preliminary analysis, we removed the MA lines that were cross-contaminated or if the depth of coverage was <20× (table 2, supplementary dataset S1: tables S2–S19, S22, Supplementary Material online).

### Phylogenetic Tree Construction

We mapped the Illumina raw reads of each ancestral line of the 15 MMR-functional strains to the *S*. Typhimurium LT2 chromosome (NCBI accession number: NC_003197.1) and used GATK-3.6 with standard hard filters to call SNPs (McKenna et al. 2010), which were then filtered using bcftools (ver. 1.8) (Danecek et al. 2021) with the following parameters: -e “AC==0 || AC==AN || F_MISSING > 0.2” -m2 -M2 -O z. vcftools (ver. 0.1.13) (Danecek et al. 2011) was then run to thin the dataset with the parameter: -thin 100 -recode. Then, we used the Ruby script convert_vcf_to_nexus.rb to convert .vcf to .nex, which can be used by PAUP (ver. 4.0a169). We used the SVDQuartets (Chifman and Kubatko 2014) module of PAUP4 (Swofford 2003) to build an unrooted tree with the parameter: evaluate all possible quartets. iTOL (ver. 6) (Letunic and Bork 2019) was then used to display and annotate the tree.

### Calculation of Population Genetic Parameters

We downloaded a total of 211 natural strains of *S. enterica*, belonging to eight serotypes, identified by the Food and Drug Administration USA from the NCBI SRA database, which were collected from 46 states of the USA (supplementary dataset S1: table S32, Supplementary Material online). We used Unicycler (ver. 0.4.8) (Wick et al. 2017) to assemble the draft genomes of all the strains, then blasted their crucial MMR genes—*mutS*, *mutL*, *mutH*, and *UvrD* with the MMR gene sequences of *S*. Typhimurium LT2. One strain (SRR8950558) was then removed due to an incomplete *mutL* gene, as we required all strains to have intact reading frames of the MMR genes. We used BWA (ver. 0.7.12) (Li and Durbin 2009) to align the raw reads of the 210 natural strains to the chromosome of *S*. Typhimurium LT2 (NCBI accession number: NC_003197.1) and calculated *π_s_*, the average pair-wise genetic distance at 4-fold degenerate sites using the following formula:

$$\pi_{s}=\frac{n}{n-1}\;\times \;\;(1-\Sigma \pi^{2})$$

where *π* is the average number of nucleotide differences per site between two DNA sequences in all possible pairs of the strains, *n* is the number of strains. The effective population size (*N_e_*) of *S*. *enterica* was calculated using the following formula, where *μ* is the mutation rate per site per cell division:

$$N_{e}=\frac{\pi_{s}}{2\mu }$$

To calculate dN/dS of the pathogenicity genes, we first retrieved the genes in *Salmonella* pathogenicity islands of *S*. Typhimurium LT2 (*marT*, *misL*, *pipA*, *rhuM*, *SopE2*, *SseC*, *SseD*, *SseE*, *SseF*, *ssrB*), as well as the homologs of other *S. enterica* strains with complete genomes available in the NCBI Genome database (supplementary dataset S1: tables S27, S28, Supplementary Material online). We then used ClustalW2 (ver. 2.1) (Larkin et al. 2007) to align the protein sequences, then used the *pal2nal.pl* script (Suyama et al. 2006) to match the aligned protein sequences with the corresponding DNA sequences in the genome and convert the amino acids to genetic codes. IQ-TREE (ver. 2.1.2) (Minh et al. 2020) was used to build the phylogenetic tree with the following parameters: -alrt 1000 -B 1000. The module codeml in PAML (ver 1.3.1) (Yang 2007) was then run to calculate the dN/dS value.

### DNA Extraction, Library Construction, and Genome Sequencing

Genomic DNA of the final evolved MA lines of each strain was extracted using the Promega Wizard® Genomic DNA Purification Kit. Quality control of DNA was performed using Qubit 3.0 and Nano-300. We constructed the libraries using Illumina Nextera® DNA Library Preparation Kit. Paired-end reads (150 bp) with insert size ∼300 bp were generated with an Illumina HiSeq2500 sequencer at the Hubbard Center for Genome Studies, University of New Hampshire, USA.

### RNA Extraction, CirSeq Library Preparation

The ancestral cells of each strain were cultured in LB broth at 37 °C on a 200 rpm shaker for 16 h. The cells were enriched in a 5430R centrifuge. Four replicates were prepared for each strain. Total RNA was extracted with the FastRNA Blue Kit (MP Biomedicals) and purified by the RNeasy Mini Kit (Qiagen). We then removed ribosomal RNA using the Illumina Ribo-Zero rRNA Removal Kit (Bacteria). The rolling-circle libraries were prepared following the protocol of Gout et al. (2017). Briefly, for each replicate of one strain, 500 ng of rRNA-depleted RNA was fragmented with the NEBNext RNase III RNA Fragmentation Module (E6146S, NEB) for 90 min at 37 °C. Then, we purified the fragmented RNA with the Oligo Clean and Concentrator Kit (Zymo Research) and used the RNA ligase 1 (M0204S, NEB) for RNA circularization. Circularized RNAs were then reverse-transcribed and amplified to cDNA. Second-strand synthesis and sequencing library preparation were then done using the NEBNext Ultra RNA Library Prep Kit for Illumina (E7530L, NEB) and the NEBNext Multiplex Oligos for Illumina (E7335S and E7500S, NEB). Agencourt AMPure XP beads (Beckman Coulter) were used during the library preparation for the clean-up and enrichment of tandem repeats that are longer than 200 bp. Another round of gel-based size selection was also performed after the library preparation to select for fragments longer than 300 bp.

### Mutation and Transcript-Error Analysis

For mutations, we ran Trimmomatic-0.32 to trim the library adaptors and low quality reads with the default parameters (ILLUMINACLIP:adaptor.fa:2:30:10 LEADING:4 TRAILING:4 MINLEN:40) (Bolger et al. 2014). We used the chromosomal sequence of *S.* Typhimurium LT2 (NCBI accession number: NC_003197.1) for the mutation and transcript-error analyses of all strains. Clean reads of the MA lines were mapped to the *S.* Typhimurium LT2 genome using BWA mem-ver. 0.7.12 (Li and Durbin 2009). We transformed the sam files to bam format using samtools-1.3.1 (Danecek et al. 2021) and duplicate reads were removed using picard-tools-2.5.0. We used HaplotypeCaller in GATK-3.6 to call SNP/indel variants with standard hard filters (McKenna et al. 2010; DePristo et al. 2011; Van der Auwera et al. 2013). We also used integrative genomics viewer to manually check mutations (Robinson et al. 2017). Context-dependent mutation rates and mutation hotspots at methylated motifs were analyzed by following Long, Sung, et al. (2015). The mutation bias *m* was calculated by *m* =  *μ*_G:C→A:T+G:C→T:A_/*μ*_A:T→G:C+A:T→C:G_, and the transition to transversion ratios (ts/tv; *n* is the total number of MA lines) with the following formula:

$$\frac{\mathrm{ts}}{\mathrm{tv}}=\frac{\sum_{1}^{n}\;transitions}{\sum_{1}^{n}\;transversions}$$

We called transcript errors following a previous study (Li and Lynch 2020). Briefly, tandem repeats were extracted to construct consensus sequences. The quality score of each consensus base call was recalculated from the raw quality score of each repeat. A probability of the erroneous consensus call of 10^−7^ or lower is required for the downstream analysis. Transcript errors were called if mismatches between consensus and reference bases were supported by <1% of reads that were mapped to the corresponding loci. Transcript errors that could be explained by genetic variations among the multiple copies of genes were further excluded as false positives.

The expected number of sites with co-occurring mutations and transcript errors in the whole genome are calculated by:

$$\mu_{G}\cdot N_{gen}\cdot \mu_{T}\cdot \bar{n}\cdot G$$

where *μ*_*G*_ is the pooled mutation rate per site per cell division of three MMR-dysfunctional strains, *N*_*gen*_ denotes the total number of cell divisions over MA experiments of the three strains, *μ*_*T*_ is the transcript-error rate per site of 15 MMR-functional strains, and $\bar{n}$ refers to the average expression level of one locus, *G* is the genome size.

To find out the association between expression levels and mutations/transcript errors, we retrieved the RNAseq data (SRR5980348–63) of *S.* Typhimurium LT2, which were all in vegetative growth, for expression level analysis. The reads were mapped to the chromosome sequence of *S*. Typhimurium LT2 using Hisat2 (ver. 2.1.0) (Kim et al. 2015), then sam files were converted to bam format using samtools-1.3.1. StringTie (ver. 2.1.5) (Kovaka et al. 2019) and Ballgown (ver. 2.5.3) (Frazee et al. 2014) were used to calculate the expression level of each gene. Finally, the expression level of each gene was calculated using the mean of the expression levels of the sample replicates.

### Testing for False Negative Mutations

The average genomic mutation rate reported here is ∼1/6 of the previously reported for *S.* Typhimurium LT2 (Lind and Andersson 2008). Artifacts resulting from the reference genome used—specifically the MA lines of non-LT2 serovars, analysis methods, batch effects of culturing media, etc., might lead to such differences.

To test if such low mutation-rate results from false negatives caused by the reference genome used (chromosome of *S.* Typhimurium LT2) or the mutation analysis method applied, we chose six strains used in this study, with complete genomes reported (the NCBI GenBank assembly accession numbers for these six strains—*S*. Agona BAA-707: GCA_000483935.1, *S*. Bareilly 9115: GCA_000487355.1, *S*. Enteritidis LJH608: GCA_003418895.1, *S*. Paratyphi A 9150: GCA_000011885.1, *S*. Typhi CT18, Ty2: GCA_000195995.1, GCA_000007545.1), and re-analyzed mutations with the GATK pipelines used in the preceding analysis, and found almost no difference (supplementary dataset S1: table S37, Supplementary Material online). The application of another consensus mutation-analysis-pipeline with the genome of *S.* Typhimurium LT2 as reference, which uses fairly simple and loose filters, also led to highly similar mutations (supplementary dataset S1: table S37, Supplementary Material online). To further test the false-negative possibility resulting from the usage of the LT2 chromosome as reference, instead of the ancestors (even though on average >90% of the MA lines’ genomes were covered with high-quality reads), we *de novo* assembled draft genomes of the 15 wild-type strains (table 2), using raw reads of the MA ancestors and Unicycler (ver. 0.4.8) with default parameters (Wick et al. 2017) (NCBI SRA, BioProject No.: PRJNA397616). We then ran all the mutation and transcript-error analyses again with the ancestral draft genomes on the MA lines or the Cir-seq samples, and found no significant differences from those using the LT2 chromosome sequence as a reference genome (supplementary dataset S1: tables S38 and S39, Supplementary Material online), adding further support to the low false negative rate of this study.

We also query if batch effects of the culturing media caused mutation rate underestimates. For each of the 15 MMR-functional strains, the MA lines were transferred in two consecutive batches with the same procedures, and the two batches did not show any significant difference in the mutation rate or spectrum, even if they were done at different time points (Paired *t*-test: *P* = 0.74; supplementary figs. S4 and S5, Supplementary Material online). Thus, the mutation rate of *S. enterica* is accurate and not technical artifacts.

All statistics were done in R 4.0.1 (R Core Team 2016). Plotting was done using Circos (Krzywinski et al. 2009) and R packages ggplot2, ggpmisc, pheatmap, and scatterplot3d.

## Supplementary Material

Supplementary data are available at *Molecular Biology and Evolution* online.

## Supplementary Material

msac081_Supplementary_Data

## Data Availability

All sequencing data in this research are available at NCBI SRA with the BioProject Number of PRJNA397616.

## References

[msac081-B1] Bateman AJ . 1959. The viability of near-normal irradiated chromosomes. Int J Radiat Biol Relat Stud Phys Chem Med. 1:170–180.

[msac081-B2] Blanc-Potard A-B , Solomon F, Kayser J, Groisman EA. 1999. The SPI-3 pathogenicity island of *Salmonella enterica*. J Bacteriol. 181:998–1004. 9922266 10.1128/jb.181.3.998-1004.1999 PMC93469

[msac081-B3] Bobay L-M , Ochman H. 2018. Factors driving effective population size and pan-genome evolution in bacteria. BMC Evol Biol. 18:153. 30314447 10.1186/s12862-018-1272-4 PMC6186134

[msac081-B4] Bolger AM , Lohse M, Usadel B. 2014. Trimmomatic: a flexible trimmer for Illumina sequence data. Bioinformatics 30:2114–2120. 24695404 10.1093/bioinformatics/btu170 PMC4103590

[msac081-B5] Chamary J-V , Parmley JL, Hurst LD. 2006. Hearing silence: non-neutral evolution at synonymous sites in mammals. Nat Rev Genet. 7:98–108. 16418745 10.1038/nrg1770

[msac081-B6] Chifman J , Kubatko L. 2014. Quartet inference from SNP data under the coalescent model. Bioinformatics 30:3317–3324. 25104814 10.1093/bioinformatics/btu530 PMC4296144

[msac081-B7] Danecek P , Auton A, Abecasis G, Albers CA, Banks E, DePristo MA, Handsaker RE, Lunter G, Marth GT, Sherry ST, et al 2011. The variant call format and VCFtools. Bioinformatics 27:2156–2158. 21653522 10.1093/bioinformatics/btr330 PMC3137218

[msac081-B8] Danecek P , Bonfield JK, Liddle J, Marshall J, Ohan V, Pollard MO, Whitwham A, Keane T, McCarthy SA, Davies RM, et al 2021. Twelve years of SAMtools and BCFtools. GigaScience 10:giab008. 33590861 10.1093/gigascience/giab008 PMC7931819

[msac081-B9] DePristo MA , Banks E, Poplin R, Garimella KV, Maguire JR, Hartl C, Philippakis AA, del Angel G, Rivas MA, Hanna M, et al 2011. A framework for variation discovery and genotyping using next-generation DNA sequencing data. Nat Genet. 43:491–498. 21478889 10.1038/ng.806 PMC3083463

[msac081-B10] Dettman JR , Sztepanacz JL, Kassen R. 2016. The properties of spontaneous mutations in the opportunistic pathogen *Pseudomonas aeruginosa*. BMC Genom. 17:27. 10.1186/s12864-015-2244-3 PMC4702332 26732503

[msac081-B11] Didelot X , Bowden R, Street T, Golubchik T, Spencer C, McVean G, Sangal V, Anjum MF, Achtman M, Falush D, et al 2011. Recombination and population structure in *Salmonella enterica*. PLoS Genet. 7:e1002191. 21829375 10.1371/journal.pgen.1002191 PMC3145606

[msac081-B12] Dillon MM , Sung W, Lynch M, Cooper VS. 2015. The rate and molecular spectrum of spontaneous mutations in the GC-Rich multichromosome genome of *Burkholderia cenocepacia*. Genetics 200:935–946. 25971664 10.1534/genetics.115.176834 PMC4512553

[msac081-B13] Dillon MM , Sung W, Sebra R, Lynch M, Cooper VS. 2017. Genome-wide biases in the rate and molecular spectrum of spontaneous mutations in *Vibrio cholerae* and *Vibrio fischeri*. Mol Biol Evol. 34:93–109. 27744412 10.1093/molbev/msw224 PMC5854121

[msac081-B14] Foster PL , Hanson AJ, Lee H, Popodi EM, Tang H. 2013. On the mutational topology of the bacterial genome. G3-Genes Genom Genet. 3:399–407. 10.1534/g3.112.005355 PMC3583449 23450823

[msac081-B15] Francioli LC , Polak PP, Koren A, Menelaou A, Chun S, Renkens I, Van Duijn CM, Swertz M, Wijmenga C, Van Ommen G, et al 2015. Genome-wide patterns and properties of de novo mutations in humans. Nat Genet. 47:822–826. 25985141 10.1038/ng.3292 PMC4485564

[msac081-B16] Frazee AC , Pertea G, Jaffe AE, Langmead B, Salzberg SL, Leek JT. 2014. Flexible isoform-level differential expression analysis with Ballgown. bioRxiv. 003665.

[msac081-B17] Fritsch C , Gout J-F, Haroon S, Towheed A, Chung C, LaGosh J, McGann E, Zhang X, Song Y, Simpson S, et al 2021. Genome-wide surveillance of transcription errors in response to genotoxic stress. Proc Natl Acad Sci U S A. 118:e2004077118. 33443141 10.1073/pnas.2004077118 PMC7817157

[msac081-B18] Galán JE . 2009. Common themes in the design and function of bacterial effectors. Cell Host Microbe 5:571–579. 19527884 10.1016/j.chom.2009.04.008 PMC2729653

[msac081-B19] Galán JE , Lara-Tejero M, Marlovits TC, Wagner S. 2014. Bacterial type III secretion systems: specialized nanomachines for protein delivery into target cells. Annu Rev Microbiol. 68:415–438. 25002086 10.1146/annurev-micro-092412-155725 PMC4388319

[msac081-B20] Gao X , Deng L, Stack G, Yu H, Chen X, Naito-Matsui Y, Varki A, Galán JE. 2017. Evolution of host adaptation in the *Salmonella* typhoid toxin. Nat Microbiol. 2:1592–1599. 28993610 10.1038/s41564-017-0033-2 PMC5705260

[msac081-B21] Gout J-F , Li W, Fritsch C, Li A, Haroon S, Singh L, Hua D, Fazelinia H, Smith Z, Seeholzer S, et al 2017. The landscape of transcription errors in eukaryotic cells. Sci Adv. 3:e1701484. 29062891 10.1126/sciadv.1701484 PMC5650487

[msac081-B22] Halligan DL , Keightley PD. 2009. Spontaneous mutation accumulation studies in evolutionary genetics. Annu Rev Ecol Evol Syst. 40:151–172.

[msac081-B23] Hensel M . 2000. *Salmonella* pathogenicity island 2. Mol Microbiol. 36:1015–1023. 10844687 10.1046/j.1365-2958.2000.01935.x

[msac081-B24] Hershberg R , Petrov DA. 2010. Evidence that mutation is universally biased towards AT in bacteria. PLoS Genet. 6:e1001115. 20838599 10.1371/journal.pgen.1001115 PMC2936535

[msac081-B25] Holt KE , Parkhill J, Mazzoni CJ, Roumagnac P, Weill FX, Goodhead I, Rance R, Baker S, Maskell DJ, Wain J, et al 2008. High-throughput sequencing provides insights into genome variation and evolution in *Salmonella* Typhi. Nat Genet. 40:987–993. 18660809 10.1038/ng.195 PMC2652037

[msac081-B26] Journet L , Cascales E, Lovett ST, Bernstein HD. 2016. The type VI secretion system in *Escherichia coli* and related species. EcoSal Plus 7:10.1128. 10.1128/ecosalplus.esp-0009-2015 PMC11575709 27223818

[msac081-B27] Kidgell C , Reichard U, Wain J, Linz B, Torpdahl M, Dougan G, Achtman M. 2002. *Salmonella typhi*, the causative agent of typhoid fever, is approximately 50,000 years old. Infect Genet Evol. 2:39–45. 12797999 10.1016/s1567-1348(02)00089-8

[msac081-B28] Kim D , Langmead B, Salzberg SL. 2015. HISAT: a fast spliced aligner with low memory requirements. Nat Methods 12:357–360. 25751142 10.1038/nmeth.3317 PMC4655817

[msac081-B29] Koch WH , Kopsidas G, Meffle B, Levine AS, Woodgate R. 1996. Analysis of chimeric UmuC proteins: identification of regions in *Salmonella typhimurium* UmuC important for mutagenic activity. Mol Gen Genet. 251:121–129. 8668121 10.1007/BF02172909

[msac081-B30] Kokubo K , Yamada M, Kanke Y, Nohmi T. 2005. Roles of replicative and specialized DNA polymerases in frameshift mutagenesis: mutability of *Salmonella typhimurium* strains lacking one or all of SOS-inducible DNA polymerases to 26 chemicals. DNA Repair 4:1160–1171. 16103022 10.1016/j.dnarep.2005.06.016

[msac081-B31] Kovaka S , Zimin AV, Pertea GM, Razaghi R, Salzberg SL, Pertea M. 2019. Transcriptome assembly from long-read RNA-seq alignments with StringTie2. Genome Biol. 20:278. 31842956 10.1186/s13059-019-1910-1 PMC6912988

[msac081-B32] Krzywinski M , Schein J, Birol I, Connors J, Gascoyne R, Horsman D, Jones SJ, Marra MA. 2009. Circos: an information aesthetic for comparative genomics. Genome Res. 19:1639–1645. 19541911 10.1101/gr.092759.109 PMC2752132

[msac081-B33] Kucukyildirim S , Long H, Sung W, Miller SF, Doak TG, Lynch M. 2016. The rate and spectrum of spontaneous mutations in *Mycobacterium smegmatis*, a bacterium naturally devoid of the postreplicative mismatch repair pathway. G3-Genes Genom Genet. 6:2157–2163. 10.1534/g3.116.030130 PMC4938668 27194804

[msac081-B34] Langridge GC , Fookes M, Connor TR, Feltwell T, Feasey N, Parsons BN, Seth-Smith HM, Barquist L, Stedman A, Humphrey T, et al 2015. Patterns of genome evolution that have accompanied host adaptation in *Salmonella*. Proc Natl Acad Sci U S A. 112:863–868. 25535353 10.1073/pnas.1416707112 PMC4311825

[msac081-B35] Larkin MA , Blackshields G, Brown NP, Chenna R, McGettigan PA, McWilliam H, Valentin F, Wallace IM, Wilm A, Lopez R, et al 2007. Clustal W and Clustal X version 2.0. Bioinformatics 23:2947–2948. 17846036 10.1093/bioinformatics/btm404

[msac081-B36] Lee H , Popodi E, Tang H, Foster PL. 2012. Rate and molecular spectrum of spontaneous mutations in the bacterium *Escherichia coli* as determined by whole-genome sequencing. Proc Natl Acad Sci U S A. 109:e2774–e2783. 22991466 10.1073/pnas.1210309109 PMC3478608

[msac081-B37] Letunic I , Bork P. 2019. Interactive tree of life (iTOL) v4: recent updates and new developments. Nucleic Acids Res. 47:w256–w259. 30931475 10.1093/nar/gkz239 PMC6602468

[msac081-B38] Li H , Durbin R. 2009. Fast and accurate short read alignment with Burrows-Wheeler Transform. Bioinformatics 25:1754–1760. 19451168 10.1093/bioinformatics/btp324 PMC2705234

[msac081-B39] Li W , Lynch M. 2020. Universally high transcript error rates in bacteria. eLife 9:e54898. 32469307 10.7554/eLife.54898 PMC7259958

[msac081-B40] Lind PA , Andersson DI. 2008. Whole-genome mutational biases in bacteria. Proc Natl Acad Sci U S A. 105:17878–17883. 19001264 10.1073/pnas.0804445105 PMC2584707

[msac081-B41] Long H , Behringer MG, Williams E, Te R, Lynch M. 2016. Similar mutation rates but highly diverse mutation spectra in ascomycete and basidiomycete yeasts. Genome Biol Evol. 8:3815–3821. 28173099 10.1093/gbe/evw286 PMC5521736

[msac081-B42] Long H , Kucukyildirim S, Sung W, Williams E, Lee H, Ackerman M, Doak TG, Tang H, Lynch M. 2015. Background mutational features of the radiation-resistant bacterium *Deinococcus radiodurans*. Mol Biol Evol. 32:2383–2392. 25976352 10.1093/molbev/msv119 PMC5009958

[msac081-B43] Long H , Miller SF, Strauss C, Zhao C, Cheng L, Ye Z, Griffin K, Te R, Lee H, Chen C-C, et al 2016. Antibiotic treatment enhances the genome-wide mutation rate of target cells. Proc Natl Acad Sci U S A. 113:E2498–E2505. 27091991 10.1073/pnas.1601208113 PMC4983809

[msac081-B44] Long H , Miller SF, Williams E, Lynch M. 2018. Specificity of the DNA mismatch repairs system (MMR) and mutagenesis bias in bacteria. Mol Biol Evol. 35:2414–2421. 29939310 10.1093/molbev/msy134 PMC6188547

[msac081-B45] Long H , Sung W, Kucukyildirim S, Williams E, Miller SF, Guo W, Patterson C, Gregory C, Strauss C, Stone C, et al 2018. Evolutionary determinants of genome-wide nucleotide composition. Nat Ecol Evol. 2:237–240. 29292397 10.1038/s41559-017-0425-y PMC6855595

[msac081-B46] Long H , Sung W, Miller SF, Ackerman MS, Doak TG, Lynch M. 2015. Mutation rate, spectrum, topology, and context-dependency in the DNA mismatch repair-deficient *Pseudomonas fluorescens* ATCC948. Genome Biol Evol. 7:262–271. 10.1093/gbe/evu284 PMC4316635 25539726

[msac081-B47] López-Cortegano E , Craig RJ, Chebib J, Samuels T, Morgan AD, Kraemer SA, Böndel KB, Ness RW, Colegrave N, Keightley PD. 2021. De novo mutation rate variation and its determinants in *Chlamydomonas*. Mol Biol Evol. 38:3709–3723. 33950243 10.1093/molbev/msab140 PMC8383909

[msac081-B48] Lostroh CP , Lee CA. 2001. The *Salmonella* pathogenicity island-1 type III secretion system. Microbes Infect. 3:1281–1291. 11755416 10.1016/s1286-4579(01)01488-5

[msac081-B49] Lou DI , Hussmann JA, McBee RM, Acevedo A, Andino R, Press WH, Sawyer SL. 2013. High-throughput DNA sequencing errors are reduced by orders of magnitude using circle sequencing. Proc Natl Acad Sci U S A. 110:19872–19877. 24243955 10.1073/pnas.1319590110 PMC3856802

[msac081-B50] Lynch M , Ackerman MS, Gout J-F, Long H, Sung W, Thomas WK, Foster PL. 2016. Genetic drift, selection and the evolution of the mutation rate. Nat Rev Genet. 17:704–714. 27739533 10.1038/nrg.2016.104

[msac081-B51] Lynch M , Sung W, Morris K, Coffey N, Landry CR, Dopman EB, Dickinson WJ, Okamoto K, Kulkarni S, Hartl DL, et al 2008. A genome-wide view of the spectrum of spontaneous mutations in yeast. Proc Natl Acad Sci U S A. 105:9272–9277. 18583475 10.1073/pnas.0803466105 PMC2453693

[msac081-B52] Lynch M , Walsh B. 2007. The origins of genome architecture. Sunderland (MA): Sinauer Associates.

[msac081-B53] McKenna A , Hanna M, Banks E, Sivachenko A, Cibulskis K, Kernytsky A, Garimella K, Altshuler D, Gabriel S, Daly M, et al 2010. The Genome Analysis Toolkit: a MapReduce framework for analyzing next-generation DNA sequencing data. Genome Res. 20:1297–1303. 20644199 10.1101/gr.107524.110 PMC2928508

[msac081-B54] Meyerovich M , Mamou G, Ben-Yehuda S. 2010. Visualizing high error levels during gene expression in living bacterial cells. Proc Natl Acad Sci U S A. 107:11543–11548. 20534550 10.1073/pnas.0912989107 PMC2895060

[msac081-B55] Minh BQ , Schmidt HA, Chernomor O, Schrempf D, Woodhams MD, von Haeseler A, Lanfear R. 2020. IQ-TREE 2: new models and efficient methods for phylogenetic inference in the genomic era. Mol Biol Evol. 37:1530–1534. 32011700 10.1093/molbev/msaa015 PMC7182206

[msac081-B56] Mukai T , Yamazaki T. 1964. The genetic structure of natural populations of *Drosophila melanogaster*. I. Spontaneous mutation rate of polygenes controlling viability. Genetics 50:1–19. 14191352 10.1093/genetics/50.1.1 PMC1210633

[msac081-B57] Ness RW , Morgan AD, Vasanthakrishnan RB, Colegrave N, Keightley PD. 2015. Extensive de novo mutation rate variation between individuals and across the genome of *Chlamydomonas reinhardtii*. Genome Res. 25:1739–1749. 26260971 10.1101/gr.191494.115 PMC4617969

[msac081-B58] Ochman H . 2003. Neutral mutations and neutral substitutions in bacterial genomes. Mol Biol Evol. 20:2091–2096. 12949125 10.1093/molbev/msg229

[msac081-B59] Pan J , Williams E, Sung W, Lynch M, Long H. 2021. The insect-killing bacterium *Photorhabdus luminescens* has the lowest mutation rate among bacteria. Mar Life Sci Technol. 3:20–27. 33791681 10.1007/s42995-020-00060-0 PMC8009600

[msac081-B60] Parkhill J , Dougan G, James KD, Thomson NR, Barrell BG. 2001. Complete genome sequence of a multiple drug resistant *Salmonella enterica* serovar Typhi CT18. Nature 413:848–852. 11677608 10.1038/35101607

[msac081-B61] R Core Team . 2016. R: A language and environment for statistical computing [Internet]. Vienna, Austria.

[msac081-B62] Robinson JT , Thorvaldsdottir H, Wenger AM, Zehir A, Mesirov JP. 2017. Variant review with the integrative genomics viewer. Cancer Res. 77:e31–e34. 29092934 10.1158/0008-5472.CAN-17-0337 PMC5678989

[msac081-B63] Roumagnac P , Weill F, Dolecek C, Baker S, Brisse S, Chinh N, Le T, Acosta C, Farrar J, Dougan G. 2006. Evolutionary history of *Salmonella* Typhi. Science 314:1301–1304. 17124322 10.1126/science.1134933 PMC2652035

[msac081-B64] Shields DC , Sharp PM, Higgins DG, Wright F. 1988. “Silent” sites in *Drosophila* genes are not neutral: evidence of selection among synonymous codons. Mol Biol Evol. 5:704–716. 3146682 10.1093/oxfordjournals.molbev.a040525

[msac081-B65] Strauss C , Long H, Patterson CE, Te R, Lynch M. 2017. Genome-wide mutation rate response to pH change in the coral reef pathogen *Vibrio shilonii* AK1. mBio 8:e01021-01017. 28830944 10.1128/mBio.01021-17 PMC5565966

[msac081-B66] Sun Y , Powell KE, Sung W, Lynch M, Moran MA, Luo H. 2017. Spontaneous mutations of a model heterotrophic marine bacterium. ISME J. 11:1713–1718. 28323279 10.1038/ismej.2017.20 PMC5584476

[msac081-B67] Sung W , Ackerman MS, Dillon MM, Platt TG, Fuqua C, Cooper VS, Lynch M. 2016. Evolution of the insertion–deletion mutation rate across the tree of life. G3-Genes Genom Genet. 6:2583–2591. 10.1534/g3.116.030890 PMC4978911 27317782

[msac081-B68] Sung W , Ackerman MS, Gout J-F, Miller SF, Williams E, Foster PL, Lynch M. 2015. Asymmetric context-dependent mutation patterns revealed through mutation–accumulation experiments. Mol Biol Evol. 32:1672–1683. 25750180 10.1093/molbev/msv055 PMC4476155

[msac081-B69] Sung W , Ackerman MS, Miller SF, Doak TG, Lynch M. 2012. Drift-barrier hypothesis and mutation-rate evolution. Proc Natl Acad Sci U S A. 109:18488–18492. 23077252 10.1073/pnas.1216223109 PMC3494944

[msac081-B70] Suyama M , Torrents D, Bork P. 2006. PAL2NAL: robust conversion of protein sequence alignments into the corresponding codon alignments. Nucleic Acids Res. 34:w609–w612. 16845082 10.1093/nar/gkl315 PMC1538804

[msac081-B71] Swofford DL . 2003. PAUP*. Phylogenetic analysis using parsimony (* and other methods). Version 4. Sunderland: Sinauer Associates.

[msac081-B72] Traverse CC , Ochman H. 2016. Conserved rates and patterns of transcription errors across bacterial growth states and lifestyles. Proc Natl Acad Sci U S A. 113:3311–3316. 26884158 10.1073/pnas.1525329113 PMC4812759

[msac081-B73] Van der Auwera GA , Carneiro MO, Hartl C, Poplin R, Del Angel G, Levy-Moonshine A, Jordan T, Shakir K, Roazen D, Thibault J, et al 2013. From FastQ data to high confidence variant calls: the Genome Analysis Toolkit best practices pipeline. Curr Protoc Bioinform. 43:11.10.11–11.10.33. 10.1002/0471250953.bi1110s43 PMC4243306 25431634

[msac081-B74] Wick RR , Judd LM, Gorrie CL, Holt KE. 2017. Unicycler: resolving bacterial genome assemblies from short and long sequencing reads. PLoS Comput Biol. 13:e1005595. 28594827 10.1371/journal.pcbi.1005595 PMC5481147

[msac081-B75] Wu K , Cheng Z-H, Williams E, Turner NT, Ran D, Li H, Zhou X, Guo H, Sung W, Liu D-F, et al 2021. Unexpected discovery of hypermutator phenotype sounds the alarm for quality control strains. Genome Biol Evol. 13:evab148. 34180992 10.1093/gbe/evab148 PMC8350357

[msac081-B76] Yang Z . 2007. PAML 4: phylogenetic analysis by maximum likelihood. Mol Biol Evol. 24:1586–1591. 17483113 10.1093/molbev/msm088

[msac081-B77] Zhao L , Gao F, Gao S, Liang Y, Long H, Lv Z, Su Y, Ye N, Zhang L, Zhao C, et al 2021. Biodiversity-based development and evolution: the emerging research systems in model and non-model organisms. Sci China Life Sci. 64:1236–1280. 33893979 10.1007/s11427-020-1915-y

[msac081-B78] Zhou Z , Inge L, Alicia TD, Sebastián D, Nabil-Fareed A, Sergeant MJ, Gemma L, Fotakis AK, Satheesh N, Stenøien HK, et al 2018. Pan-genome analysis of ancient and modern *Salmonella enterica* demonstrates genomic stability of the invasive Para C lineage for millennia. Curr Biol. 28:2420–2428.e10. 30033331 10.1016/j.cub.2018.05.058 PMC6089836

