# Supplementary material for: Rates of Mutations and Transcript Errors in the Foodborne Pathogen Salmonella enterica subsp. enterica
Source: Mol Biol Evol. 2022 Apr 21;39(4):msac081. doi: 10.1093/molbev/msac081 (PMC9040049; doi:10.1093/molbev/msac081)
Supplement: msac081_Supplementary_Data [file msac081_supplementary_data.zip › Supplementary Material.docx]

**Supplementary Information: Rates of mutations and transcript errors** **in the foodborne pathogen *Salmonella enterica* subsp. *enterica***

**Authors:** Jiao Pan^1,2,#^, Weiyi Li^3,#^, Jiahao Ni^1,#^, Kun Wu^1^, Iain Konigsberg^4^, Caitlyn Patterson^3^, Clayton Tincher^3^, Colin Gregory^3^, Xia Zhou^1^, Thomas G. Doak^3,5^, Heewook Lee^6^, Yan Wang^1^, Xiang Gao^7^, Michael Lynch^8^, Hongan Long^1,2,*^

**Affiliations:**

^1^Institute of Evolution and Marine Biodiversity, KLMME, Ocean University of China, Qingdao, Shandong Province, China 266003

^2^Laboratory for Marine Biology and Biotechnology, Qingdao Pilot National Laboratory for Marine Science and Technology, Qingdao, China 266237

^3^Department of Biology, Indiana University, Bloomington, IN, USA 47405

^4^Division of Biomedical Informatics & Personalized Medicine, Department of Medicine, University of Colorado, Aurora, CO, USA 80045

^5^National Center for Genome Analysis Support, Indiana University, Bloomington, IN, USA 47405

^6^School of Computing and Augmented Intelligence, Arizona State University, Tempe Arizona USA 85281

^7^State Key Laboratory of Microbial Technology, Microbial Technology Institute, School of Life Science, Shandong University, No. 72 Binhai Road, Qingdao, Shandong Province, China, 266237

^8^Biodesign Center for Mechanisms of Evolution, Arizona State University, Tempe, Arizona, USA 85281

*Author for Correspondence:

Hongan Long, Institute of Evolution and Marine Biodiversity, KLMME, Ocean University of China, Qingdao, Shandong Province, China 266003; Laboratory for Marine Biology and Biotechnology, Qingdao Pilot National Laboratory for Marine Science and Technology, Qingdao, China 266237; phone +86 532 82031723; email: [longhongan@ouc.edu.cn](mailto:longhongan@ouc.edu.cn)

Fig. S1

Fig. S2

Fig. S3

Fig. S4

Fig. S5

**Other supplementary materials for this manuscript include the following:**

Dataset S1


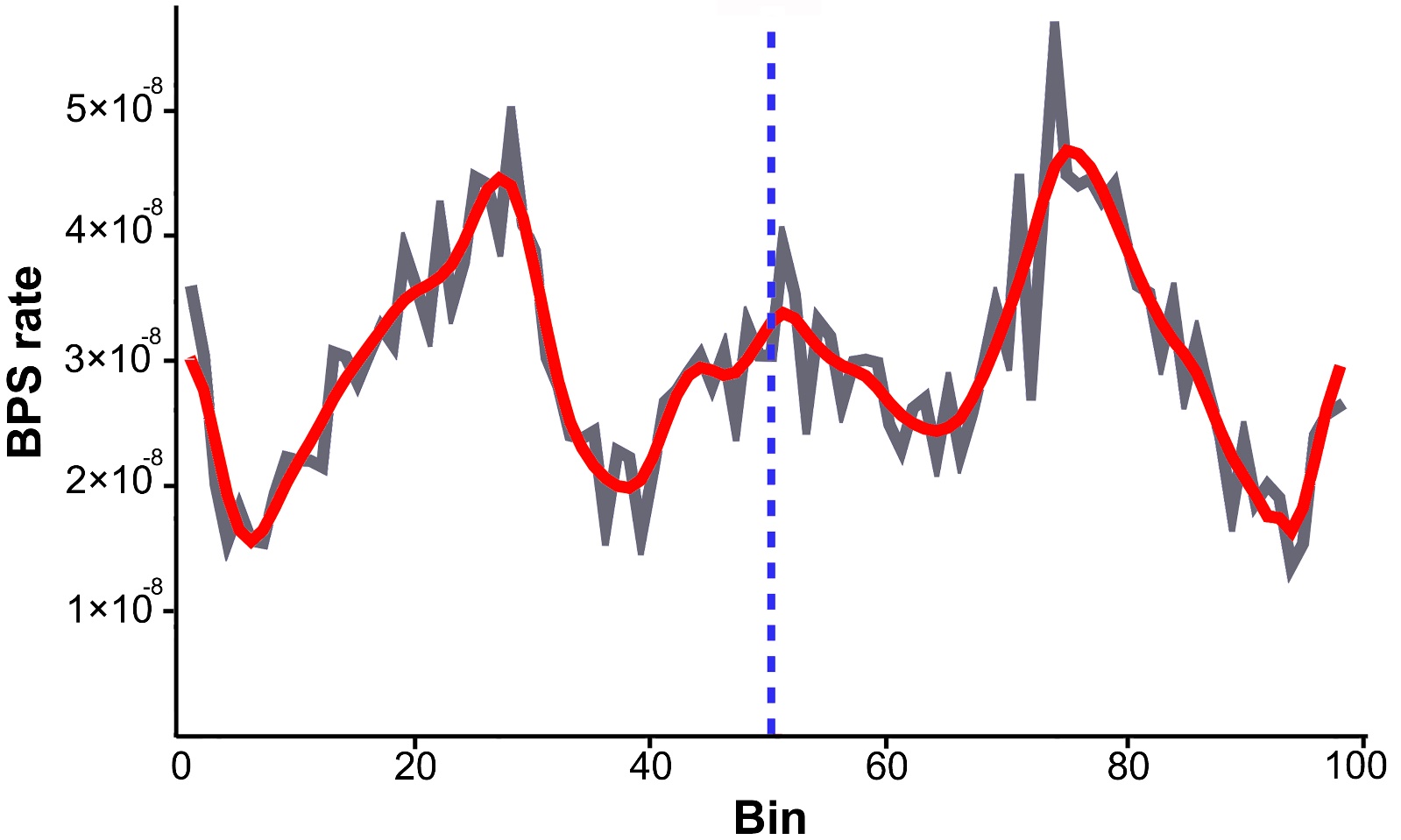


**Fig. S1.** **The mutation topology inferred by base substitutions accumulated in the three MMR-dysfunctional LT2 strains (in 50-kb bins).** The grey curve shows distribution of mutation rate directly plotted; the red curve shows distribution after wavelet transformation (Foster, et al. 2013); the replication terminus is shown by the blue dashed line; the leftmost of the curves starts from the origin of replication.


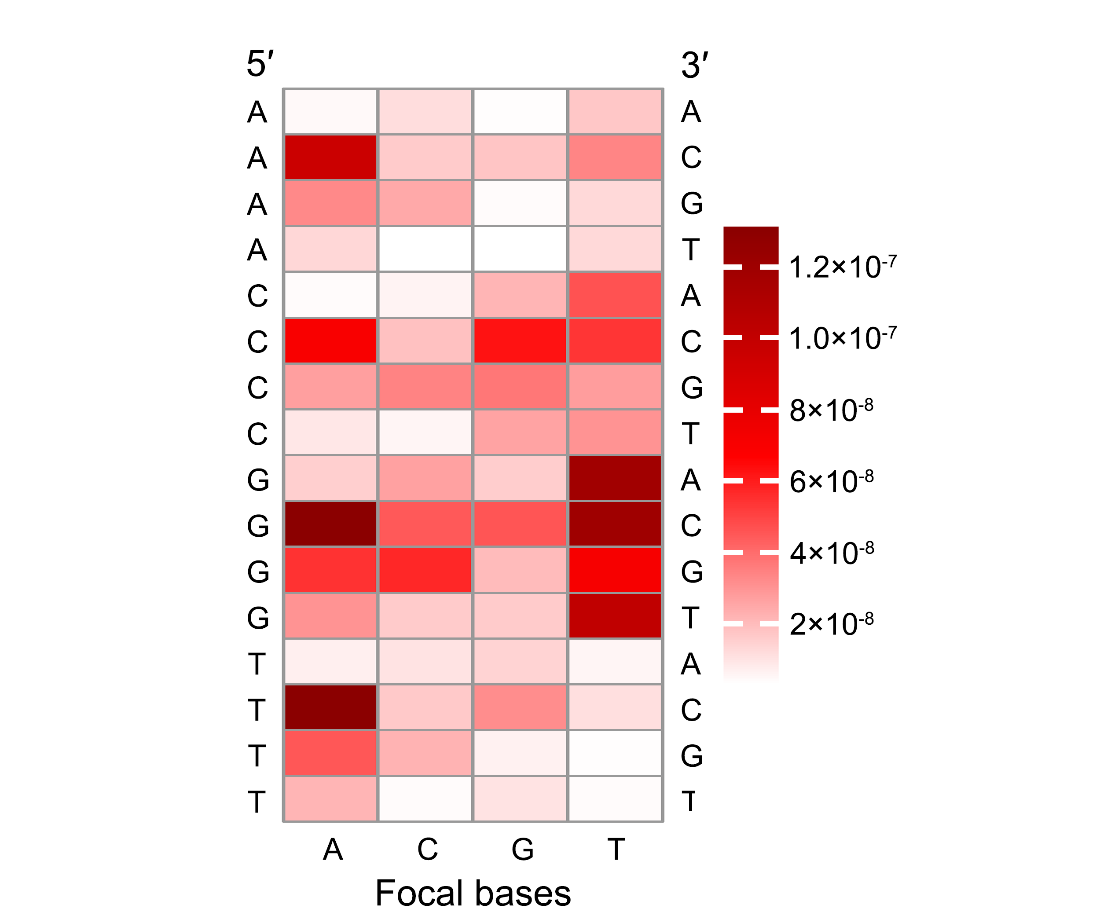


**Fig. S2. Context-dependent mutation rate.** The heat-map shows mutation numbers with different nucleotide contexts, based on mutations of the three MMR-deficient LT2 strains.


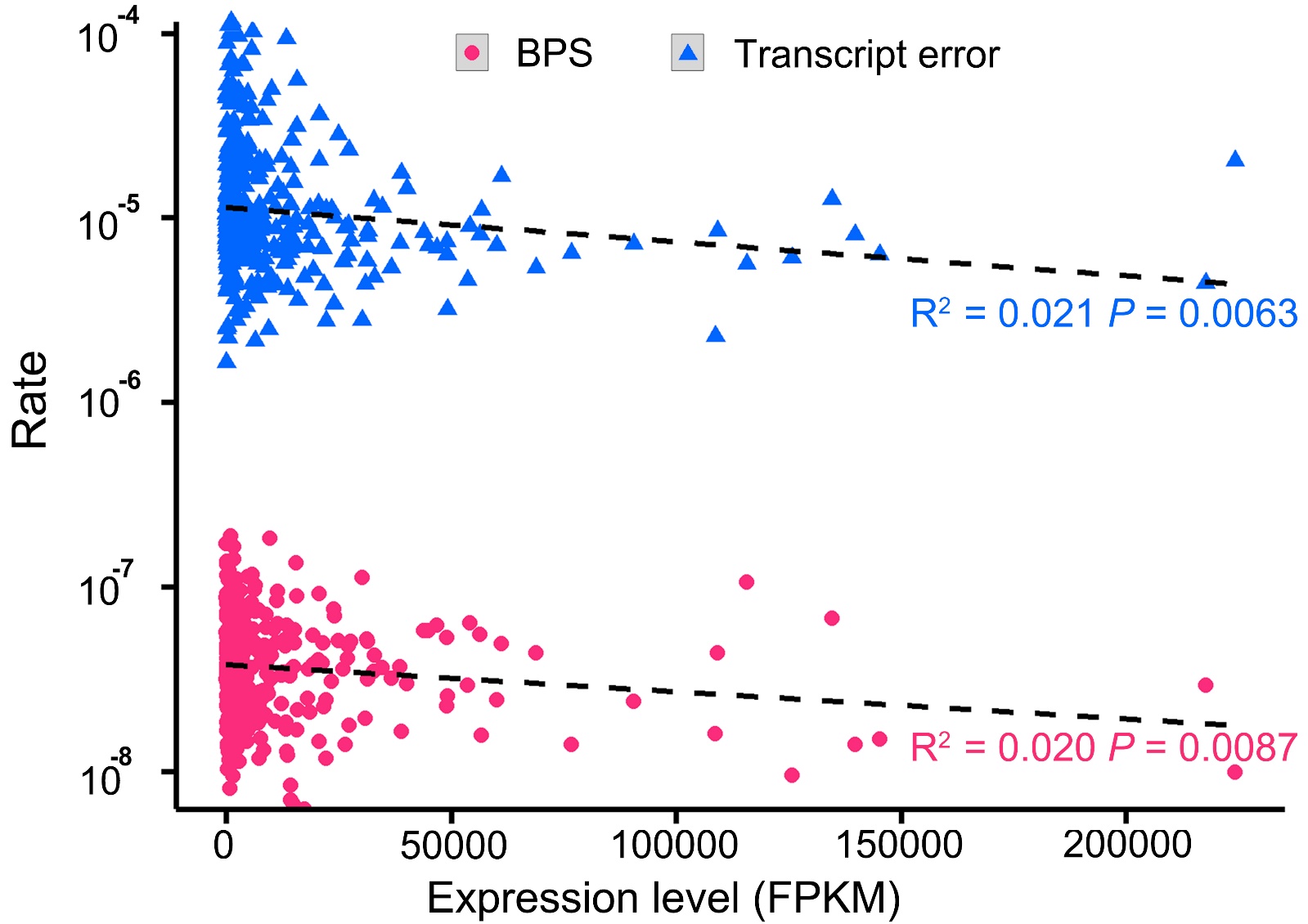


**Fig. S3. The relationship between expression levels of different genes and the rates of mutations or transcript errors** (FPKM**:** fragments per kilobase per million mapped fragments); BPS are base substitutions pooled from the three MMR-dysfunctional strains (y = –7.42–1.46×10^-6^ x); transcript errors are from all 15 MMR-functional strains (y = –4.94–1.85×10^-6^ x).


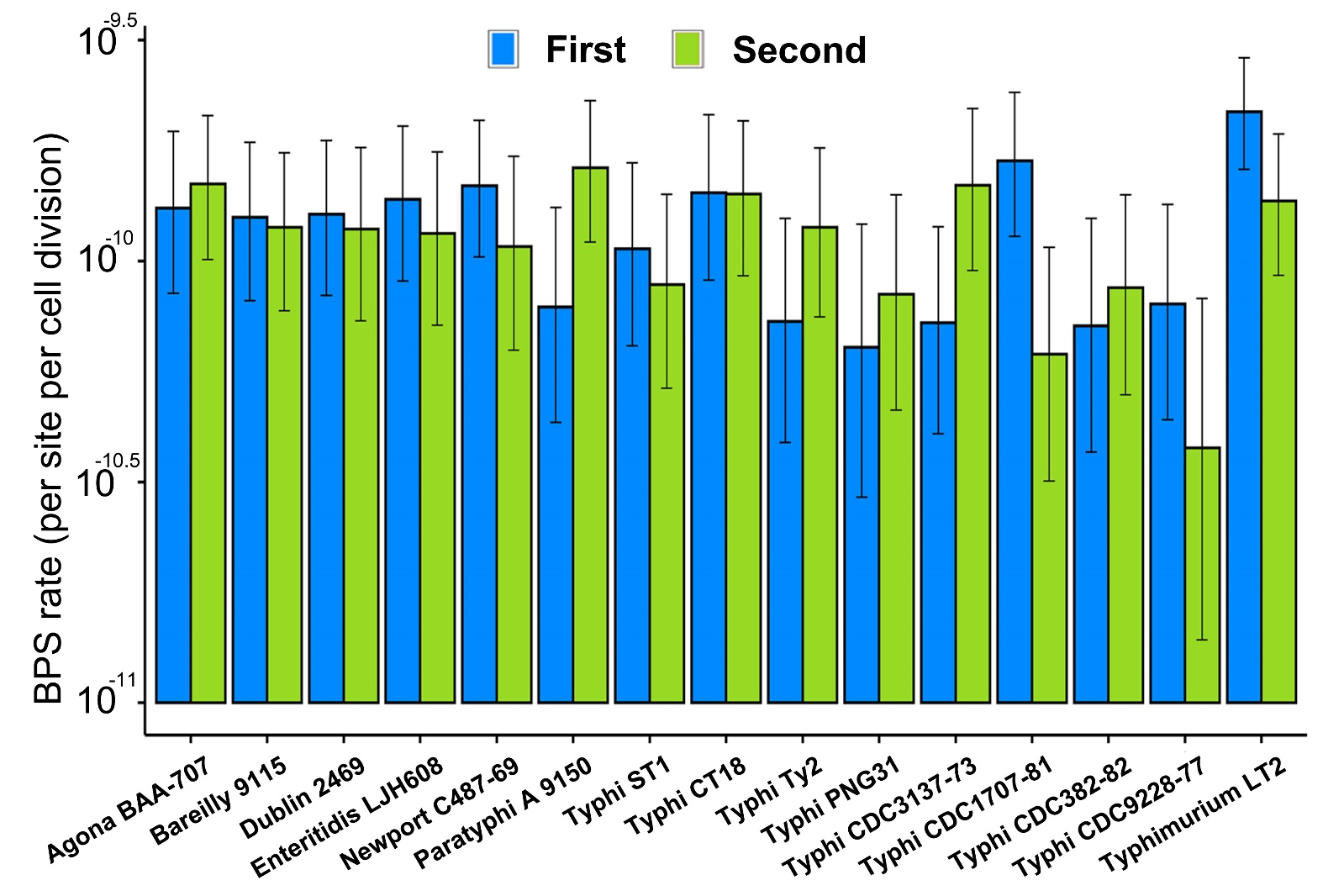


**Fig. S4. The different BPS mutation rates of two contiguous batches of MA lines for each of the 15 MMR-functional strains of *S. enterica*.** Error bars are 95% Poisson confidence intervals.


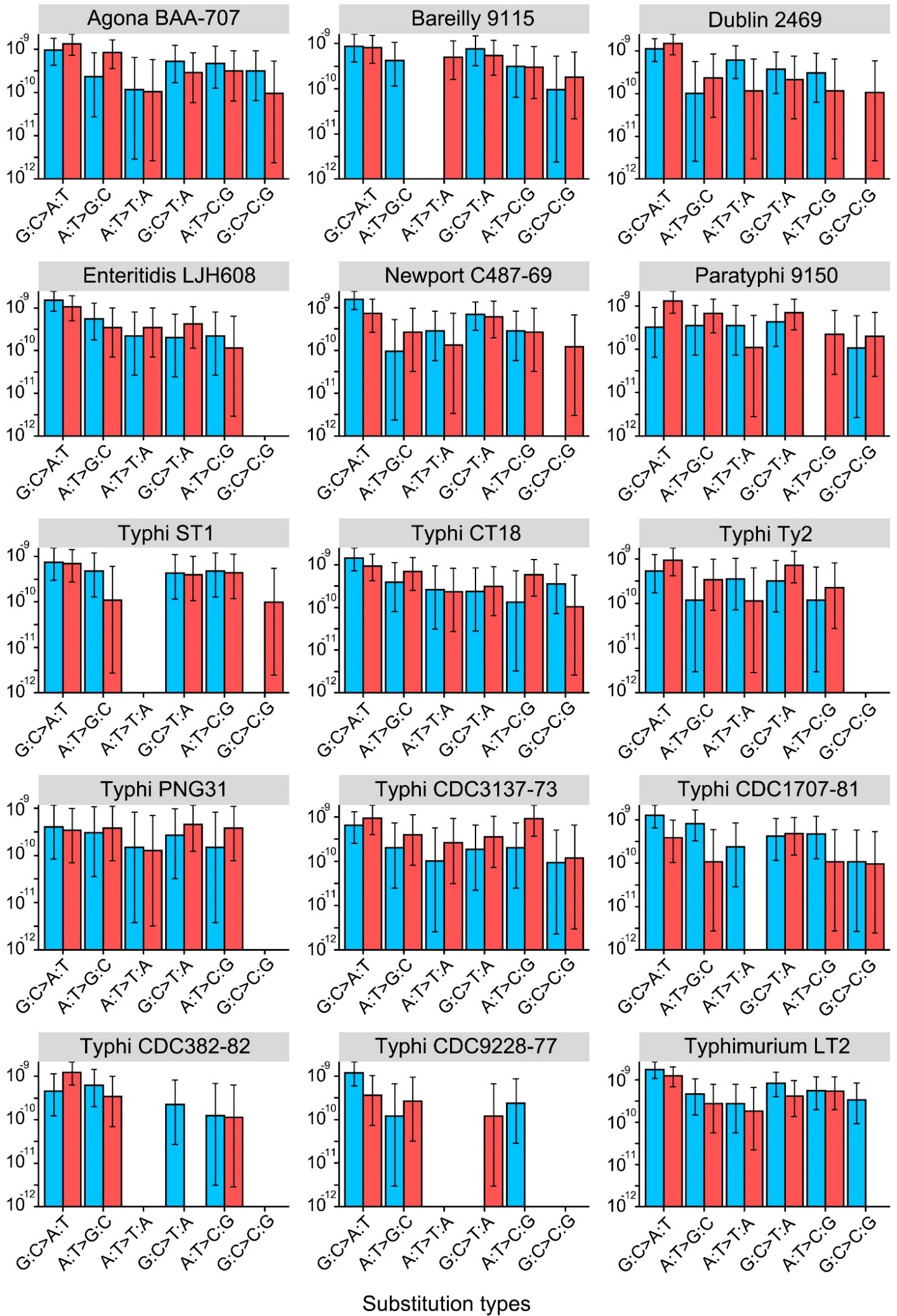


**Fig. S5.** **The different mutation spetra of two contiguous batches of MA lines for the 15 MMR-functional strains of *S. enterica*.** Y-axis numbers are in the unit of per site per cell division. Error bars are 95% Poisson confidence interval.
